# Flexible sensor concept and an integrated collision sensing for efficient human-robot collaboration using 3D local global sensors

**DOI:** 10.3389/frobt.2023.1028411

**Published:** 2023-04-06

**Authors:** Aquib Rashid, Ibrahim Alnaser, Mohamad Bdiwi, Steffen Ihlenfeldt

**Affiliations:** Department of Cognitive Human Machine Systems, Fraunhofer Institute for Machine Tools and Forming Technology, Chemnitz, Germany

**Keywords:** collision avoidance, human–robot collaboration, intrusion distance, sensor concept, distance sensors

## Abstract

Human-robot collaboration with traditional industrial robots is a cardinal step towards agile manufacturing and re-manufacturing processes. These processes require constant human presence, which results in lower operational efficiency based on current industrial collision avoidance systems. The work proposes a novel local and global sensing framework, which discusses a flexible sensor concept comprising a single 2D or 3D LiDAR while formulating occlusion due to the robot body. Moreover, this work extends the previous local global sensing methodology to incorporate local (co-moving) 3D sensors on the robot body. The local 3D camera faces toward the robot occlusion area, resulted from the robot body in front of a single global 3D LiDAR. Apart from the sensor concept, this work also proposes an efficient method to estimate sensitivity and reactivity of sensing and control sub-systems The proposed methodologies are tested with a heavy-duty industrial robot along with a 3D LiDAR and camera. The integrated local global sensing methods allow high robot speeds resulting in process efficiency while ensuring human safety and sensor flexibility.

## 1 Introduction

Traditional industrial robots better complement human workers with their large range and high payload capabilities. These capabilities are required in multiple manufacturing processes. Furthermore, Gerbers et al. (2018), Huang et al. (2019), Liu et al. (2019), and Zorn et al. (2022) also proposed a human–robot collaborative disassembly as a means of sustainable production. More than 95% of the total robots installed in the world between 2017 and 2019 are traditional industrial robots (Bauer et al., 2016). Moreover, there is a gradual increase in the single human–single robot collaborative processes (IFR, 2020).

Touchless and distance-based collision avoidance systems are required to enable traditional industrial robots to collaborate efficiently with humans. Increased efforts toward e-mobility and sustainability have opened new challenges in the waste disposal sectors. For example, the shredding of batteries and cars decreases engineering production value. Disassembly, however, can help further save engineering and energy costs while reducing carbon emissions. Full automation of the disassembly would require a large amount of data for AI engines, which can be collected with an intermediate solution of a constant human–robot collaboration. Ensuring operational efficiency and varied requirements for disassembly processes requires new collision sensing methodologies.

Current safety standards aim to completely stop the robot before the human comes in contact. These standards enabled fenceless robot cells, where safety sensors like the laser scanner/2D LiDAR sensor are installed to ensure a protective separation distance (PSD) between the human and robot as follows:

$$\begin{array}{c} PSD\left(t_{0}\right)\geq {C+S}_{h}+S_{r}+S_{s}+Z_{R}+Z_{S}, \end{array}$$ (1)

where C is the linear intrusion distance inside the sensor field of view, after which a detection is triggered. $S_{h}\&\;S_{r}$ are the distances covered by the human and robot before the actuating system reacts to the signal from the sensing system. $S_{s}$ is the distance covered by the robot before stopping while being controlled by the actuating system. $Z_{R}\;and\;Z_{S}$ are the inaccuracies in position estimation from the robot and sensor, respectively. This strategy is termed speed and separation monitoring (SSM) and is applicable for presence detection-based sensors with occasional operator presence.

Eq. 1 can be used inversely (Byner et al., 2019) when the actual separation distance (ASD) between the robot and human is known to determine the maximum safety robot velocity of the robot ( $v_{r}^{safe}$ ) as follows:

$$\begin{array}{c} v_{r}^{safe}\leq \sqrt{v_{h}^{2}+{\left(T_{r}a_{s}\right)}^{2}-2a_{s}\left(C-ASD\right)}-T_{r}a_{s}-v_{h} \end{array},$$ (2)

where $T_{r}$ is the reaction time of the actuation system, $a_{s}$ is the maximum negative deceleration of the robot, and $v_{h}$ is the expected human velocity. This approach is termed dynamic speed and separation monitoring (DSSM).

**FIGURE 1:**
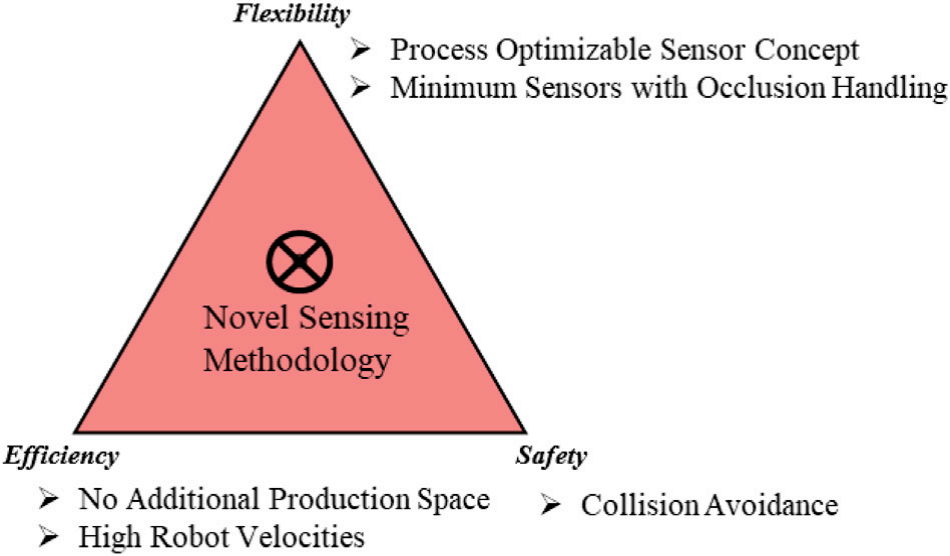
Research target.

The main focus of this work is to develop a novel sensing methodology, which can be used in the context of traditional industrial robots with constant human presence. The state of the art is evaluated toward this goal with three main parameters, as shown in Figure 1. Agile production requires a flexible sensor concept, which can be adjusted to the need of the process. Furthermore, as each process may require different levels of complexity and human intervention, the sensing methodology should be flexible and scalable while ensuring occlusion handling with minimum sensors. Two main conditions are considered for efficiency. Previously set-up robotic cells may have limited production space as resources. Moreover, the operational efficiency of the process should be achieved by high robot velocities. Finally, collision avoidance would be ensured for the complete human body, which requires 3D sensing.

The main contribution of this work is the methodologies proposed in the design and implementation of the sensor concept for a human–robot collaboration with traditional industrial robots. These contributions are highlighted as follows:

1) Co-existence cell design, which discusses the LiDAR-based sensor concept with limited resources, variable need for shared space, and utilization of the entrance area for prior detection.
2) An efficient method to estimate the intrusion distance and reaction time parameters of a collision avoidance system for speed and separation monitoring.

## 2 State of the art

2D LiDAR-based sensing approaches have been discussed. This approach approximates the human position based on a cylindrical model (Som, 2005). The safety approach implemented here is based on Tri-mode SSM (Marvel, 2013), which includes not only PSD (the stop area) but also slow and normal speed areas. Nevertheless, the approach results in low operational efficiency due to the constant presence of humans in the slow area. Byner et al. (2019) ensured higher efficiency by proposing dynamic speed and separation monitoring. Nevertheless, the 2D LiDAR approach limits the applicability in the constant operator presence scenario, where the upper limbs are not detected. Human upper limbs can move at twice the speed of the estimated human velocity (Weitschat et al., 2018). The 3D LiDAR approach with a higher vertical field of view and accuracy than the 2D LiDAR approach and fixed field of view (FOV) 3D depth cameras. Moreover, fixed FOV-based 3D depth cameras require additional production space to capture the complete robot workspace area, as discussed by Morato et al. (2014).

Flacco et al. (2012) proposed efficient and high robot velocities for a limited workspace area. The depth camera is installed outside of the robot body, looking toward the human workspace area. The approach, however, suffers from occlusion from a large traditional robot. Moreover, no method is proposed to ensure compliance with the safety standards as no intrusion distance measurements are provided.

The single sensor-based approach by Kuhn and Henrich.,(2007) projected an expanding convex mesh from a virtual robot model on images. The minimum distance was estimated by performing a binary search until an unknown object intersects the projected hull. Similar to Flacco, the approach is not applicable for large traditional industrial robots as the sensor concept would require additional space to ensure covering the large robot body.

Two important research problems have been identified:

1) A generic sensor concept needs to be proposed, which discusses the 2D or 3D LiDAR sensor concept from the aspects of flexibility and occlusion handling.
2) Furthermore, means to measure the intrusion distance for 3D cameras on the robot body need to be discussed.

Sensor concept and design aspects have been proposed by Flacco and De Luca (2010), addressing presence and detection-based sensors. Nevertheless, they are not applicable for 2D LiDAR with a large traditional robot causing the main occlusion. Moreover, the intrusion distance measurement for sensors on the robot body is extended from our previous work (Rashid et al., 2022).

## 3 Flexible and efficient sensor concepts for LiDAR and 3D camera

**FIGURE 2:**
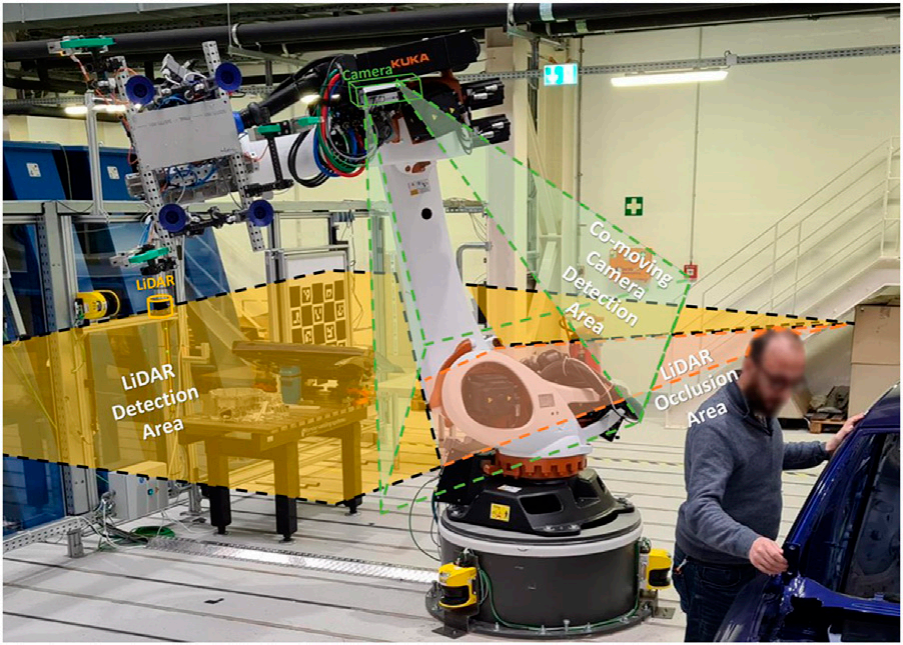
Proposed flexible and efficient sensor system.

The work proposes a novel local global sensing framework, which comprises 1) a flexible global (LiDAR-based) sensor concept and 2) an efficient local (3D cameras) intrusion distance measurement method. The integrated approach provides an efficient and flexible collaboration with traditional industrial robots using the novel local global sensing methodology. The local-global sensing in the previous work used a stationary LiDAR and camera sensor (Rashid et al., 2021). This work further introduces co-moving cameras as local sensors to ensure safety from grippers and objects gripped, as illustrated in Figure 2.

### 3.1 Flexible global sensor concept

This section discusses an occlusion-aware sensor concept applicable to 2D and 3D LiDAR. The sensor concept is discussed by proposing a standardized co-existence cell model in 2D based on three parameters. These parameters are maximum robot reach r, space available toward the entry area e, and the number of entry sides n.

**FIGURE 3:**
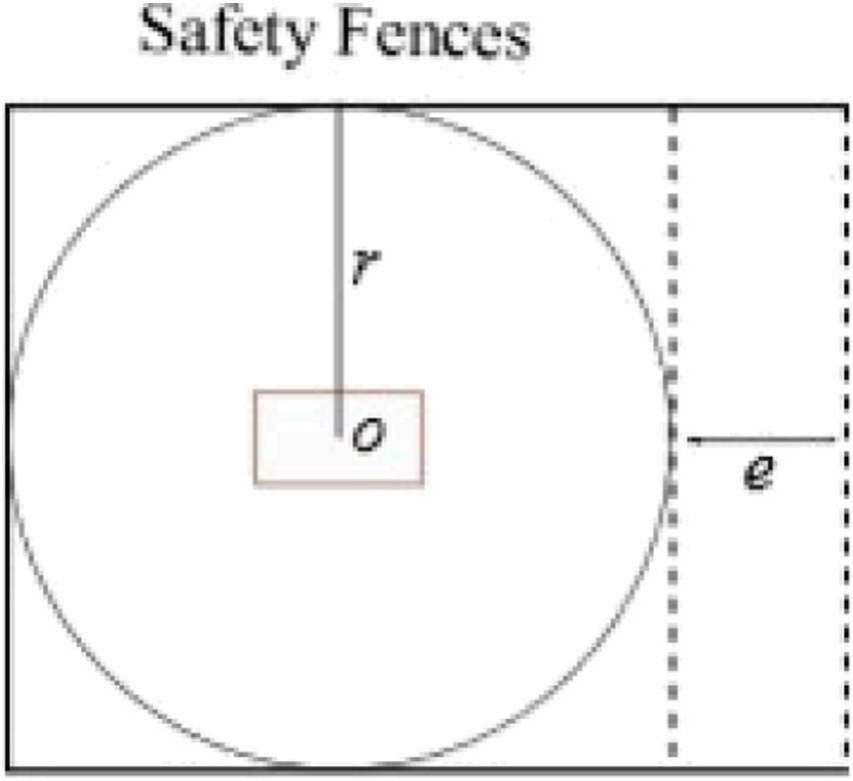
Simplified co-existence cell.

Occlusion with a single LiDAR sensor can be caused due to static objects in the field of view or a dynamic robot body. For the LiDAR sensor placed without any orientation, the robot base link results in the most evident occlusion in the cell. The green circle represents the LiDAR sensor, and the robot base link is represented with a rectangle with maximum length and width (*l* x *w*). The base rectangle is placed at the center of the robot workspace, which is enclosed by three sides (n = 1) with safety fences. The entrance area e is assumed to be free from any static occlusions. A ray from LiDAR, represented by a dotted red line, which when intercepted, results in entry occlusion, is termed a boundary ray, as shown in Figure 3.

For the sensor position in scenario A, illustrated in Figure 3, occlusion is constrained by the safety fence, thus having a lower risk of possible human collision. Moreover, for scenario B, the occlusion is unconstrained toward the entrance area and is at higher risk of a possible collision. Unconstrained occlusions are avoided by allowing LiDAR placement only on the adjacent entrance walls. Furthermore, the boundary ray polar coordinates are used to set constraints on robot motion. These measures allow safety by design. The constrained occlusion caused by the robot base requires to be mathematically formulated.

**FIGURE 4:**
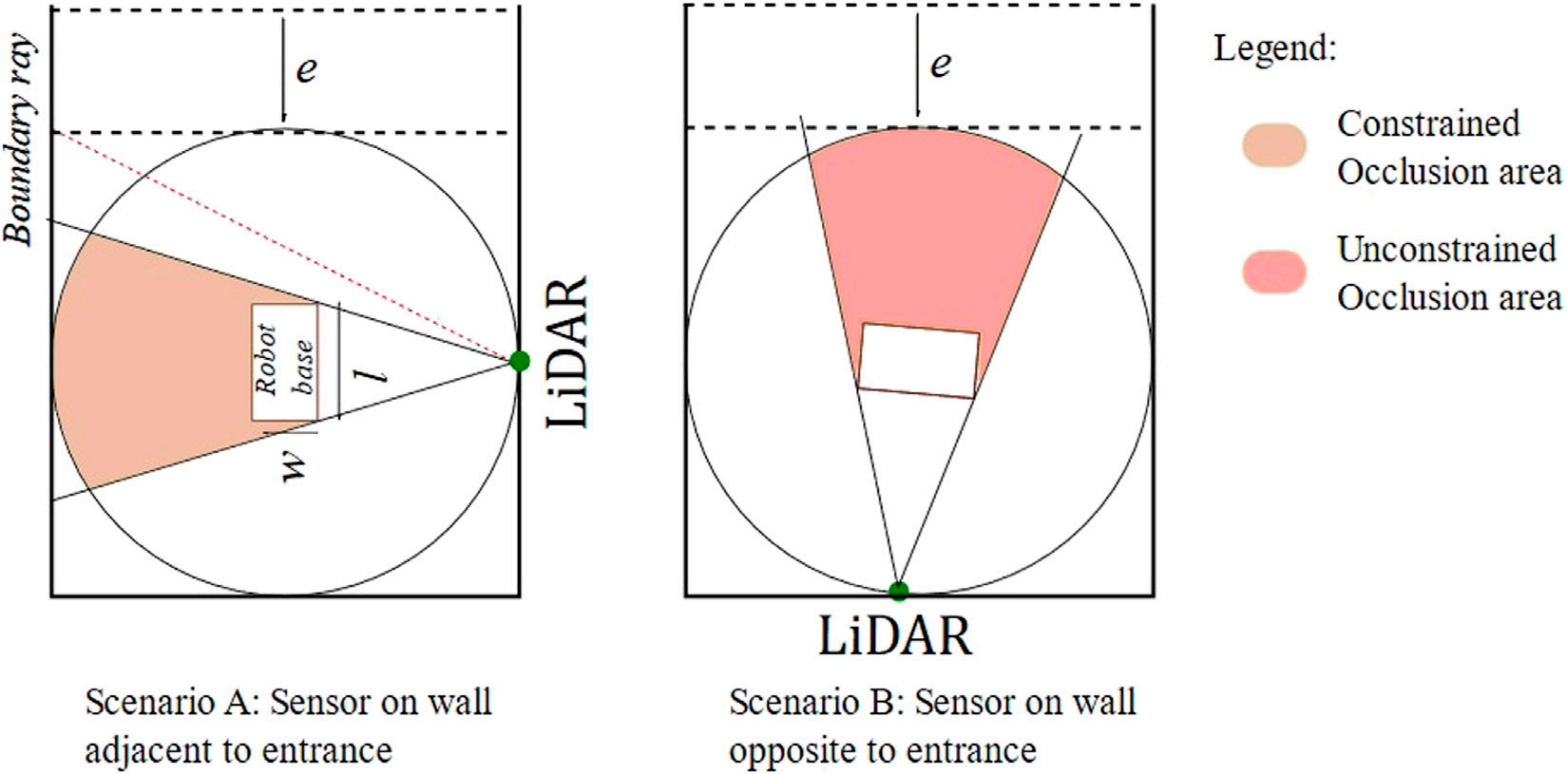
Different types of occlusions.

In the 6D pose for the 360° HFOV LiDAR, no orientations are assumed to exist for the robot reference frame. Any yaw or pitch orientations would result in non-uniform coverage of the production area. Moreover, 3D LiDAR comprises multiple 2D laser channels, which rotate at a certain orientation, as illustrated in Figure 4. The height parameter has a direct relationship with the area of the circle with the radius r1 . The circle represents the blind spot in the 2D floor space of the co-existence cell and can be expressed as follows:

$$r1=h*\tan\left(\alpha \right),$$ (3)

thus lowering the height of LiDAR results in a decreased blind spot area. On the other hand, increasing the height of the LiDAR, to a specific extent, results in increasing the number of rays falling inside the robot cell. The higher number of rays corresponds to a higher accuracy of human localization estimations. An optimal height would be related to average worker heights and sensor vertical resolution. The risks from the blind spot area can be reduced by ensuring that the human is localized minimum by the 0° measuring plane. This leaves the XY plane, on which the Y-axis is constrained to avoid unconstrained occlusion. Thus, only 1D degree of freedom is available for the LiDAR sensor concept. Nevertheless, this 1D is enough to cover a variety of process requirements, while incurring no additional production space.

**FIGURE 5:**
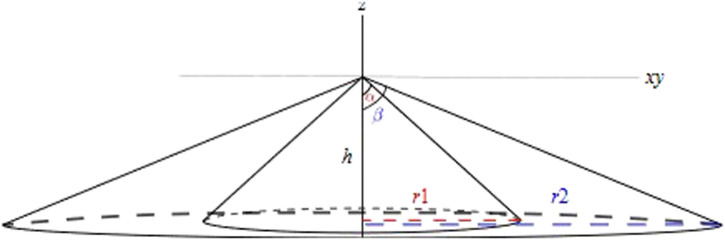
LiDAR sensor concept.

Let the LiDAR sensor be placed at A*,* representing the middle of the safety fence. Maximum occlusion is caused when the robot base is perpendicular to the global sensor. This occlusion area is represented by an area of polygon IHFEDC, as illustrated in Figure 5. The occluded area, in this configuration, can be computed in robot parameters, by drawing perpendicular OP on AC and joining OC, as shown in Figure 5. The occluded area CPEFHI is computed by first computing its half area, constituting the area of ∆ AOC and sector BOC. Then, removing half of the base rectangle and ∆ AGD gives a symmetric one-sided constrained occlusion area.

The sector BOC inscribes the same arc BC as that of BAC. This results in the angle (α) at the sector BOC, being twice the angle ( θ ) at the sector BAC. Using this, the area of the sector BOC can be defined as follows:

$$\begin{array}{c} Area\;BOC=\frac{2\theta }{360}*\pi r^{2} \end{array},$$ (4)

where r represents the maximum reach of the robot used.

Furthermore, the area of ∆ AOC can be computed as follows:

$$\begin{array}{c} ∆AOC=\frac{1}{2}\left(OP*AC\right). \end{array}$$ (5)

Using the property of isosceles triangle ∆ AOC,

$$\begin{array}{c} AC=2AP \end{array}.$$ (6)

Sides AP and OP can be expressed as follows:

$$\begin{array}{c} AP=PC=r*\cos\theta , \end{array}$$ (7)

$$\begin{array}{c} OP=r*\sin\theta . \end{array}$$ (8)

Substituting Eqs 6, 7, and 8 in Eq. 5, we obtain the following equation:

$$\begin{array}{c} ∆AOC=\frac{1}{2}\left(\left(r*\sin\theta \right)*2\left(r*\cos\theta \right)\right). \end{array}$$ (9)

Using Eqs 4 and 9, the area of BAC can be defined as follows:

$$\begin{array}{c} \mathrm{Area}\;\;\mathrm{of}\;\mathrm{BAC}=\frac{2\left(\theta \right)}{360}*\pi r^{2}+\frac{1}{2}\left(\left(r*\sin\theta \right)*2\left(r*\cos\theta \right)\right). \end{array}$$ (10)

The symmetric half occlusion area can be computed using Eq. 10, by removing the area of the robot body and ∆AGD as follows:

$$\begin{array}{c} \mathrm{Area}\;of\;BJEDC=\mathrm{Area}\;\mathrm{BAC}-\frac{1}{2}\left(\mathrm{Area}\;of\;\mathrm{Rectangle}\;EFHD\right)-\mathrm{Area}∆AGD. \end{array}$$ (11)

Using Eqs 9–11, we get

$$\mathrm{Area}\;\;of\;BJEDC=\frac{2\left(\theta \right)}{360}*\pi r^{2}+\frac{1}{2}\left(\left(r*\sin\theta \right)*\left(2r*\cos\theta \right)\right)-\frac{1}{2}\;\left(l*w\right)-\frac{1}{2}\;\left(\frac{l}{2}*\left(r-\frac{w}{2}\right)\right).$$ (12)

Eq. 12 can be used to compute the overall occlusion area IHFEDC as follows:

$$\mathrm{Area}\;of\;IHFEDC=2\;\left(\mathrm{Area}\;of\;BJEDC\right)\;\begin{array}{c} =2\;\left(\frac{2\left(\theta \right)}{360}*\pi r^{2}+\frac{1}{2}\left(\left(r*\sin\theta \right)*\left(2r*\cos\theta \right)\right)-\frac{1}{2}\;\left(l*w\right)-\frac{1}{2}\;\left(\frac{l}{2}*\left(r-\frac{w}{2}\right)\right)\right). \end{array}$$ (13)

Eq. 1 gives a generalized equation to compute the occlusion area, with the distance between the sensor and robot *r*, robot base link dimensions, and occlusion angle ( 2θ ) with the robot body.

For a specific sensor position on the safety fence in a co-existence cell model, the overall occlusion area can also be defined by representing the value of angle **∠**GAD (θ) as follows:

$$\begin{array}{c} \theta =\tan^{-1}\left(\frac{GD}{AG}\right)=\tan^{-1}\left(\frac{\frac{l}{2}}{r-\frac{w}{2}}\right). \end{array}$$ (14)

Substituting Eq. 13 into Eq. 14, the overall generalized calculation of the occlusion area, with the sensor at the middle point of the co-existence cell fence, as shown in Figure 5, is given by the following:

$$\begin{array}{c} Area\;of\;IHFEDC=2*\;\left(\begin{array}{c} \frac{2\left(\tan^{-1}\left(\frac{\frac{l}{2}}{r-\frac{w}{2}}\right)\right)}{360}*\pi r^{2}+\\ \frac{1}{2}\left(\begin{array}{c} \left(r*\sin\left(\tan^{-1}\left(\frac{\frac{l}{2}}{r-\frac{w}{2}}\right)\right)\right)*\\ \left(2r*\cos\left(\tan^{-1}\left(\frac{\frac{l}{2}}{r-\frac{w}{2}}\right)\right)\right) \end{array}\right)-\\ \frac{1}{2}\;\left(l*w\right)-\frac{1}{2}\;\left(\frac{l}{2}*\left(r-\frac{w}{2}\right)\right) \end{array}\right). \end{array}$$ (15)

**FIGURE 6:**
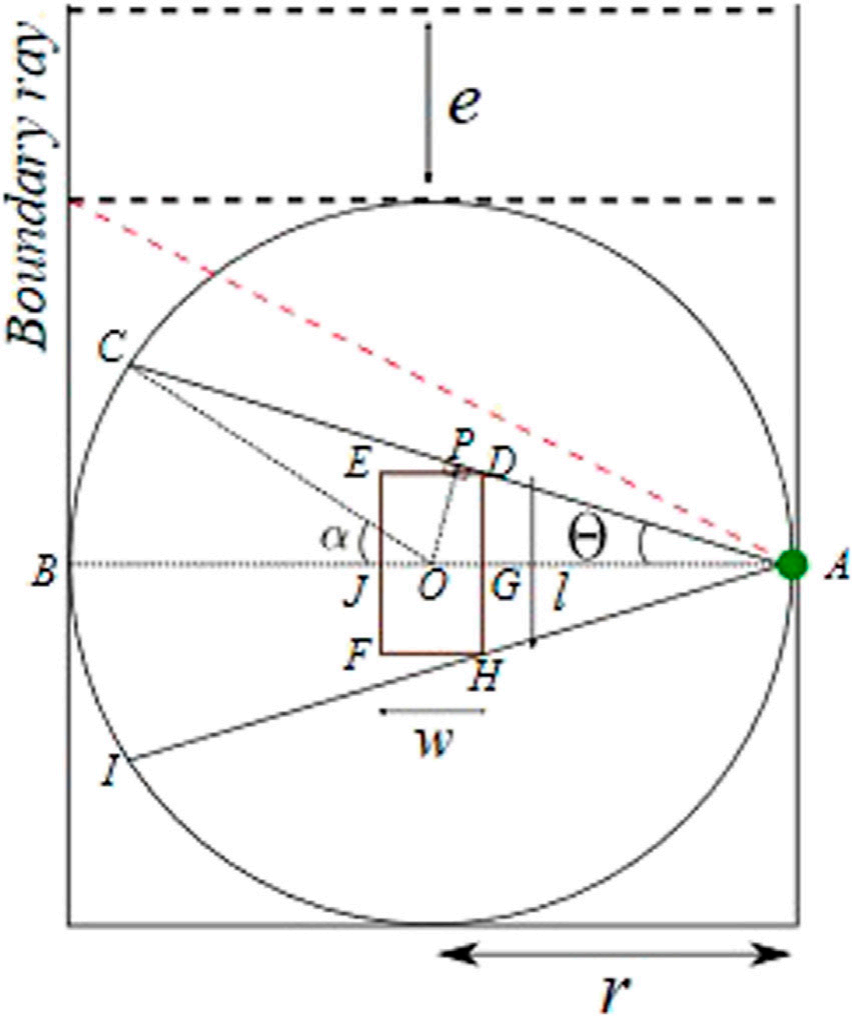
Constrained occlusion with sensor at middle of fence.

Similarly, for the sensor placed at the entrance corner start position, as illustrated in Figure 6, the radius r2 can be computed as half of the length of the diagonal of the square as follows:

$$\begin{array}{c} r2=\sqrt{2}r \end{array}.$$ (16)

The aforementioned Eq. 16 is substituted in 13, resulting in the overall occluded area ( Area of IHFEDC ) at the start position as follows:

$$=2\;*\;\left(\begin{array}{c} \frac{2\left(\tan^{-1}\left(\frac{\frac{l}{2}}{\sqrt{2}r-\frac{w}{2}}\right)\right)}{360}*\pi r^{2}\\ +\frac{1}{2}\left(\left(r*\sin\left(\tan^{-1}\left(\frac{\frac{l}{2}}{\sqrt{2}r-\frac{w}{2}}\right)\right)\right)*\left(2r*\cos\left(\tan^{-1}\left(\frac{\frac{l}{2}}{\sqrt{2}r-\frac{w}{2}}\right)\right)\right)\right)\\ -\frac{1}{2}\;\left(l*w\right)-\frac{1}{2}\;\left(\frac{l}{2}\;*\left(\sqrt{2}r-\frac{w}{2}\right)\right) \end{array}\right),$$

where *w* and *l* represent the width and length of the base link. It is evident that the constrained occlusion for the scenario with LiDAR at the entrance is minimum. Nevertheless, the optimal position of the LiDAR (global) sensor could be adjusted based on process requirements and available resources.

The 1D variation of LiDAR between the start and midpoint of the fence fulfills varied perception requirements for varied processes. The final placement of LiDAR would divide the overall workspace into 1) constrained occlusion and 2) shared workspace. The occupancy of these workspaces is checked in the real-time collision avoidance system, using polar coordinate limits. Extrinsic calibration between the robot and LiDAR is assumed to be known (Rashid et al., 2020). Finally, the constrained occlusion area can further be covered using a local 3D camera on the robot body, facing toward the shadow area. The local collision avoidance setup is already discussed in our previous work (Rashid et al., 2021).

### 3.2 Intrusion distance and reaction time estimation for co-moving local sensors

The proposed method provides the intrusion distance estimation for co-moving local sensors on the robot body. This method comprises three main steps. The first step involves setting up an external intruding object, which can be detected from the presence detection algorithm for local sensing. This is followed by multiple controlled robot experiments at a defined angular velocity. The data coming from the robot joints and sensing detections are recorded in an external processing system. The final step involves offline processing of the recorded data, with some a priori data as input to provide safety parameters as output. These sub-steps are discussed in detail in Section 3.2.2.

#### 3.2.1 Controlled experiments’ overview from illustrations

**FIGURE 7:**
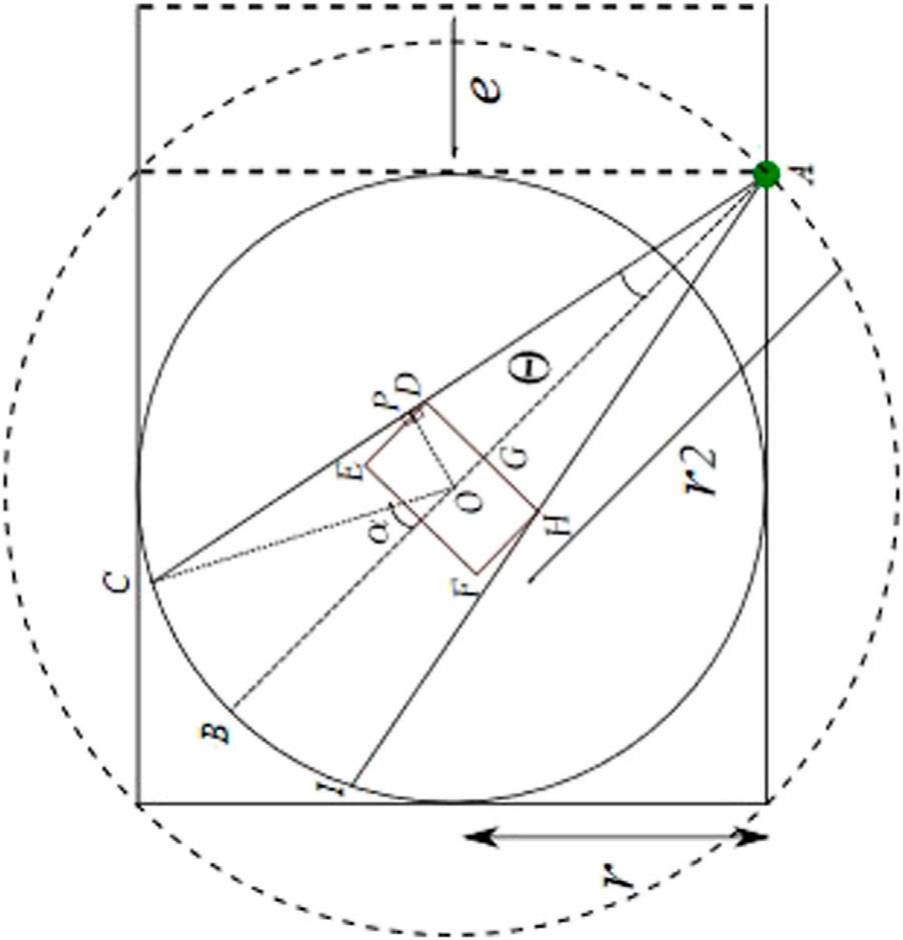
Constrained occlusion with sensor at the entrance.

A simplistic concept for the estimation of the parameter for local sensing can be understood in Figure 7. The intruding object of height I is placed in the robot work cell at a distance of a vector. A 3D camera is mounted on the robot body with reference frame S and a field of view Θ, as illustrated in Section 1 of Figure 7. A robot trajectory with the tool center point velocity of $v_{j}$ is performed, ensuring that the intruding object is piercing almost perpendicularly into the sensor field of view, as illustrated in section II of Figure 7. The known system and intruding object features ensure the pre-estimation of the expected detection (d_x), where the sensor field of view first touches the intruding object. However, the sensor data being processed in an external system results in a delay. Thus, the actual detected (d_a) is flagged at the future position, giving an estimate for intrusion distance (C), as illustrated in part III of Figure 7. The flagged detection issues a stop signal from the external processing system to the robot controller. The stopping trigger (tr) is perceived by the start of unplanned deceleration in the external system capturing the robot velocities, as illustrated in part IV of Figure 7. The delay in communication to an external processing system is assumed to be negligible. Moreover, most robot manufacturers provide communication interfaces running at 250–1000 Hz. Finally, the motion of the sensor is assumed to result in linear displacement of the intruding object into the field of view.

#### 3.2.2 Setting up of the intruding object

A simple cardboard box placed on a stand is used as an intruding object. The setup of this intruding object requires positioning the intruding object at a known position with respect to the robot reference frame. The second step of the robot experiment is illustrated in Figure 7. The detection of the intruding object, which is not part of the environment with respect to a moving sensor on the robot body, requires unknown object detection based on local sensing (Rashid et al., 2021).

#### 3.2.3 Offline data processing method for intrusion distance and reaction time measurements

This step involving the offline post-processing of the recorded data is detailed in the following algorithm. The developed software tool parses through multiple iterations at a specific robot velocity. The expected detection (d_x) for a specific set of positions is taken and processed sequentially. In a single iteration for a known intruding object position, the robot position is searched in angular joint coordinates, where the detection flag is active. The corresponding joint angular position JointAngle_(d_a)^i is compared to the actual detection (∝_(d_x)) to estimate the intrusion distance. The time stamp at the position of the flagged detection is recorded (t_(d_x)). The joint angular velocities are searched for a deceleration trigger for which the corresponding time stamp is captured (t_tr). The difference between the two time stamps provides an estimate of controller reaction time.

Algorithm Reaction time and Intrusion distance

**Input**: Time-stamped Joint Angles, Joint Angular velocity, and Detection Flag over multiple iterations ( i ) and over a complete set represented by j,k,l,or event

{ ${time}_{t}^{i},{JointAngle}_{j}^{i},{Joint\%age}_{k}^{i},,\propto_{d\_x}$ }
**Output**: Reaction time (B) of robot in ms; $Intrusion\;distance\;\propto_{in}$ in mm
**Algorithm**:
A ←{ $\left({time}_{t}^{i},{JointAngle}_{j}^{i},{Joint\%age}_{k}^{i},\Theta_{eventExpected}\right)$ }
B ← ∅
**While** A ≠ ∅ **do/**/Search for the event expected position if ${JointAngle}_{j}^{i}$ ≥ $\propto_{d\_x}$

$t_{d\_x}$ ← ${time}_{te}^{i}$ //Save time stamp
**if** eventDetect= 1//Actual event detected

$\propto_{in}={JointAngle}_{d\_a}^{i}-\propto_{d\_x}$

**While** ${Joint\%age}_{n}^{i}$ ≠ ∅//Search for deceleration trigger **if** ${Joint\%age}_{k-9}^{i}$ > ${Joint\%age}_{k-8}^{i}$ >… ${Joint\%age}_{k}^{i}$

$t_{tr}$ ← ${time}_{ta}^{i}$

B ← $t_{tr}$ – $t_{d\_x}$ **return** B; $\propto_{in}$

## 4 Experimental setup for efficient intrusion distance estimation

**FIGURE 8:**
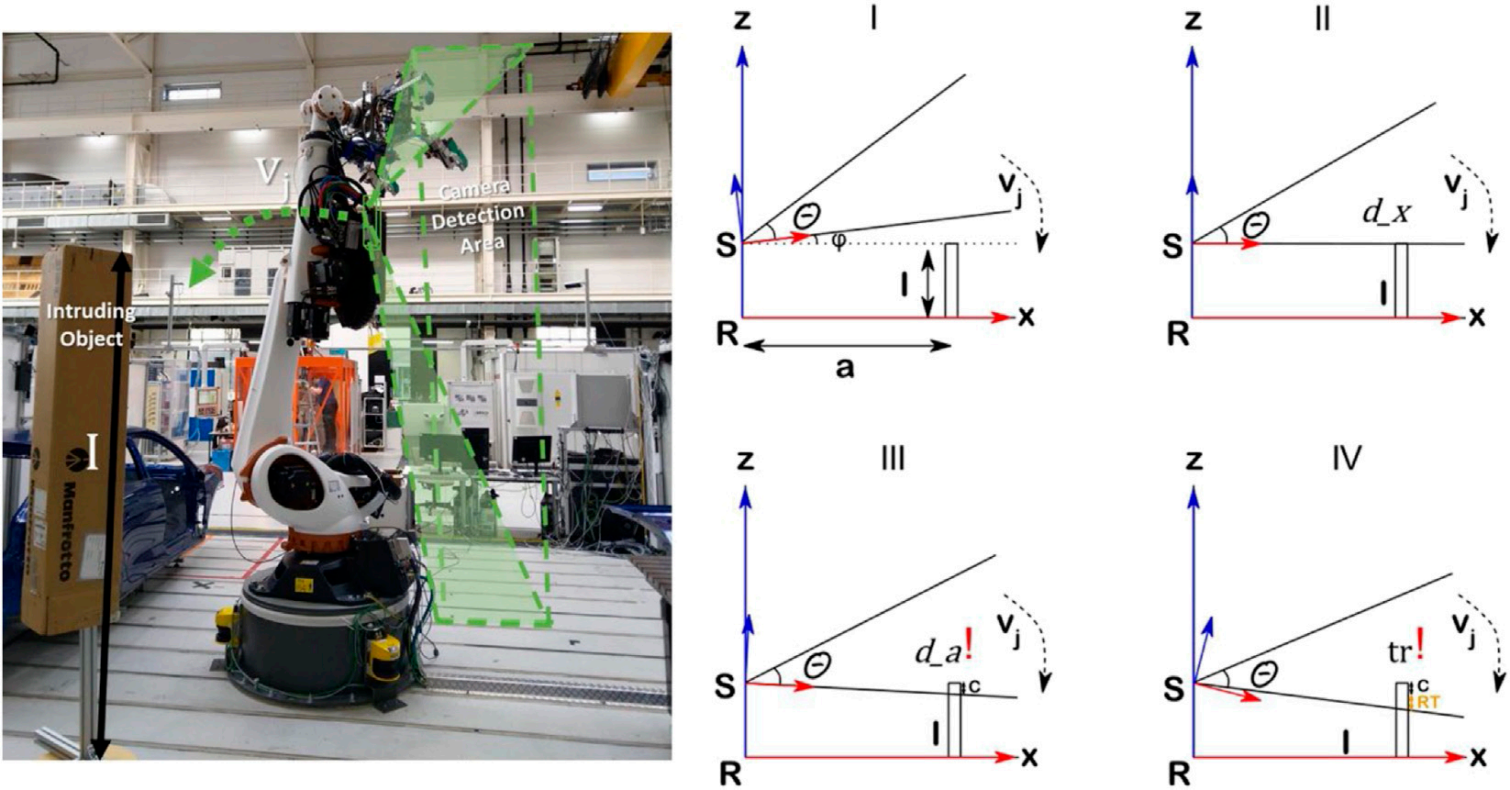
Data Capturing experiment for safety parameter estimation.

The experimental setup comprises a stereo camera connected to a processing system over a USB3 connection. The processing system is connected over Ethernet to a robot controller. In this work, ZED1 from Stereo Labs is used as a 3D camera. A heavy-duty industrial robot Kuka KR180 with a range of 2.9 m is used, as illustrated in Figure 8. The KRC4 robot controller is used, which allows 250 Hz UDP communication with an external computer. Joint angular displacement along with a tool center point (TCP) in the base coordinate is provided at the external processing system. In order to compare the intrusion distance of local sensing (Schrimpf, 2013) with that of global sensing (Morato et al., 2014), an identical processing system is used with Intel i7-6700K CPU, Nvidia TITAN Xp GPU, and 12 GB GDDR5 memory.

At 4 ms, as for Kuka communication speed, the robot joint values are used to estimate the position of the local sensor on the robot. A cardboard box is placed in a robot cell of 4 x 4 m. The position of the intruding object is estimated by moving the TCP to the top of the object. More accurate estimations can be performed by using ArUco markers (Garrido-Jurado et al., 2014) on the intruding object. The intruding position for the known sensor is estimated with a ±10 mm precision.

### 4.1 Experimental results

**FIGURE 9:**
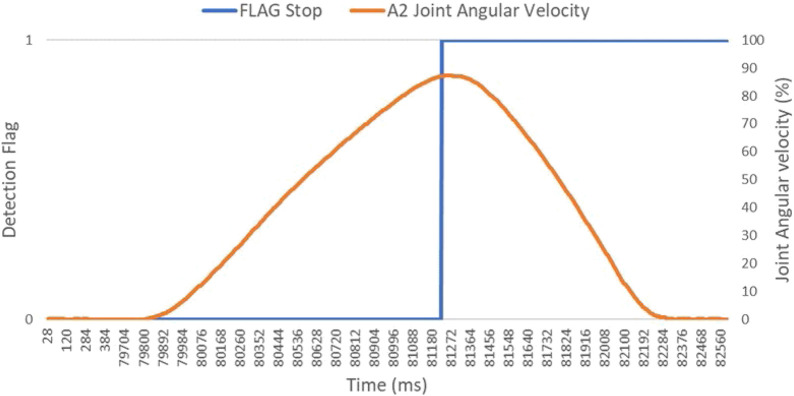
Complete trajectory for collision sensing and stop trigger for a single iteration.

The real-time experiment with more than five iterations for a single robot velocity is captured for statistical variations. The robot velocities need not be running at a constant velocity before an event, compared to the state of the art (Rashid et al., 2022). This can be challenging for achieving a high robot velocity with a large sensor field of view. This work rather uses a constant accelerating profile, with specific top velocities. An important aspect here is to capture linear robot velocities, which are comparable to or higher than the nominal human speed (1.6 m^2^). A constant acceleration profile can be seen in Figure 9. The figure gives a dual vertical graph for joint angular velocity and a detection flag for A2 joint-based motion. The approx. linear velocity for the sensor mounted on A3 amounts to be

$$\begin{array}{c} v_{l}=r*\varphi /t, \end{array}$$ (17)

where r is the approximate length of the A2 link, which is 1.35 m for Kuka KR180. φ is angular displacement in radians, and t is the total time duration in seconds, Thus, the linear velocity was found to be 1.12 m/s2.

**FIGURE 10:**
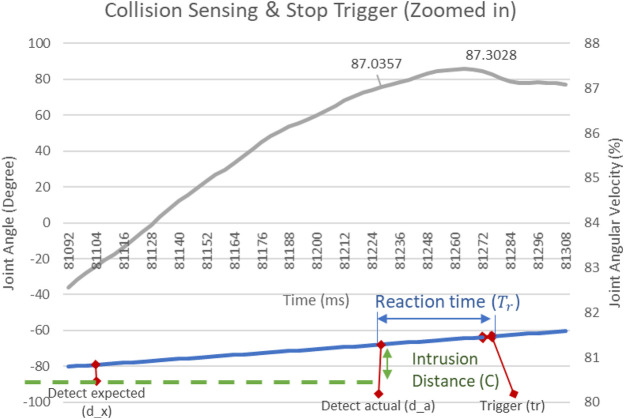
Zoomed in trajectory for intrusion distance and reaction time estimation.

Figure 9 gives a zoomed-in view into the recorded velocity profile for the experiment data from Figure 10, with a target speed of 87% of A2 joint speed. The sensing flag and deceleration trigger are observed at 81,228 and 81,276 timestamps, respectively. The intrusion distance and reaction time is measured in spatial and temporal dimensions.

**FIGURE 11:**
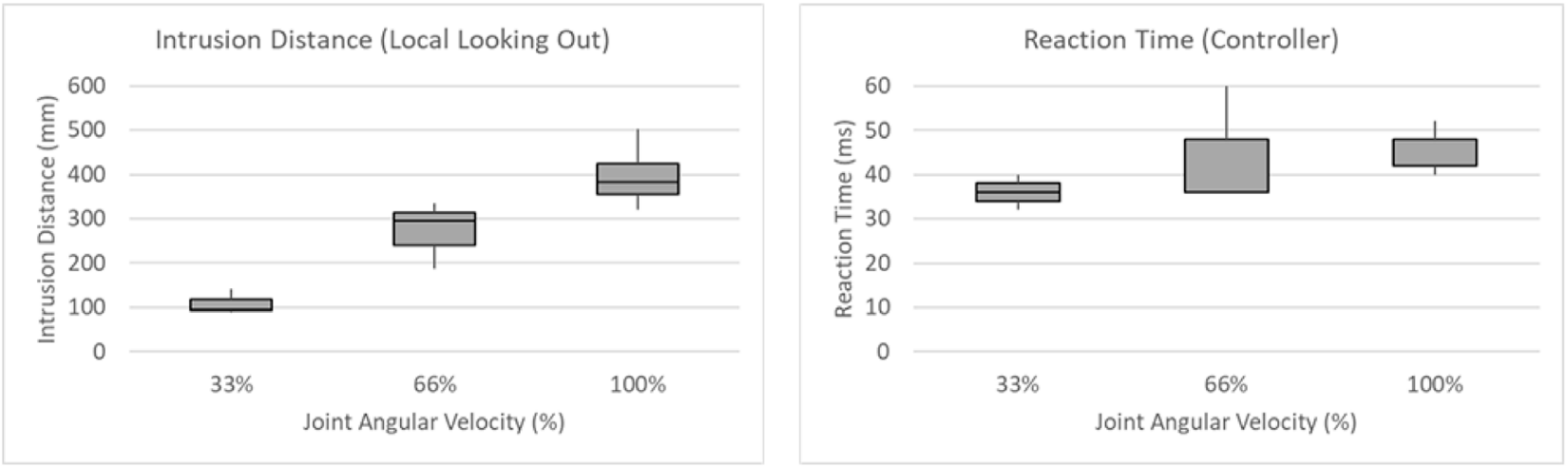
Box plot on multiple iterations at multiple robot velocities.

The implementation of local sensing is discussed in Mandischer et al. (2022). The multiple iterations on three robot velocities are processed to estimate the worst-case intrusion distance and reaction time, as illustrated in Figure 11. The worst-case reaction time is calculated as 60 ms, which is comparable to the state-of-the-art calculation of 56 ms and 40 ms (Schrimpf, 2013) for different robot controllers. The worst-case intrusion distance captured at multiple iterations and velocities equals to be 502 mm.

### 4.2 Discussion on the efficient and flexible constant human–robot collaboration with traditional industrial robots

**FIGURE 12:**
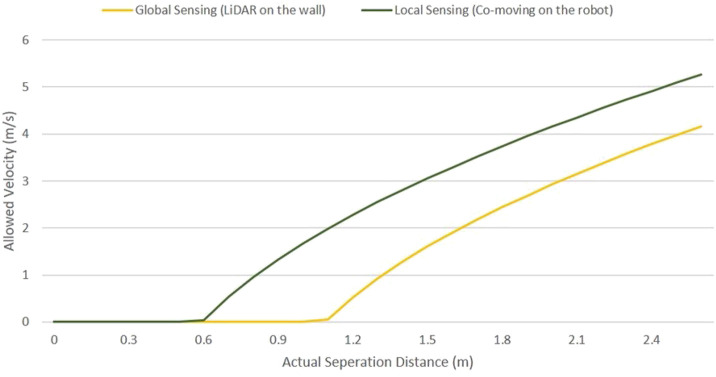
Safety distance and robot velocity for local (co-moving) and global collision sensing.

The method proposed allows safety distance measurement for co-moving or dynamic local sensors on the robot body. The method can be used by system integrators or safety sensor developers, aiming to use distance-based sensors for an efficient collaborative system. Plotting the worst-case intrusion distance of local sensing with global sensing in Eq. 2, we obtain two different relations between the separation distance and maximum allowed robot velocity, as illustrated in Figure 12. The co-moving local sensor or dynamic local sensing is more efficient, allowing higher robot speed within the proximity human operator. However, the co-moving local sensor is only relevant for the safety of the tool or object in the robot’s hand. The LiDAR sensor on the wall, acting as a global sensor, ensures safety from the complete robot body without the constrained occlusion area. This occlusion area can be constantly monitored based on the LiDAR sensor concept. Furthermore, safety from design can be implemented using the LiDAR sensor concept by allotting human workspace away from the constrained occlusion. Future steps would include determining an optimal combination of different local (co-moving/dynamic (Mandischer et al., 2022) or static (Rashid et al., 2021)) and global sensing (Rashid et al., 2021) systems for a given agile or disassembly process in a safety digital twin.

## 5 Conclusion

The work proposes a method for not only utilizing distance-based sensors for an efficient and flexible collision avoidance system for a human–robot collaboration. The global sensor concept with LiDAR addresses a minimalistic and reduced complexity approach while addressing the occlusion from the robot body. Furthermore, an efficient method is proposed that simultaneously determines the intrusion distance and reaction time for 3D cameras on the robot body and robot controller, respectively. The work proposed can be used to compute safety parameters for a wide variety of distance-based sensors on robot bodies. These methodologies can be implemented for lightweight industrial robots or co-bots. Increased efforts toward resource efficiency and sustainability will require human–robot collaboration. The methodologies proposed will enable the development of close-proximity human–robot collaboration.

## Data Availability

The raw data supporting the conclusion of this article will be made available by the authors, without undue reservation.
