# Erratum: Flexible sensor concept and an integrated collision sensing for efficient human-robot collaboration using 3D local global sensors

**DOI:** 10.3389/frobt.2023.1228130

**Published:** 2023-06-13

**Authors:** Frontiers Production Office

**Affiliations:** Frontiers Media SA, Lausanne, Switzerland

**Keywords:** collision avoidance, human–robot collaboration, intrusion distance, sensor concept, distance sensors

Due to a production error, there was an error in the figures as published. The correct Figures 2–9, Figures 11, 12 appear below.

The article title was revised to read *“Flexible sensor concept and an integrated collision sensing for efficient human-robot collaboration using 3D local global sensors”.*

The publisher apologizes for these mistakes. The original version of this article has been updated.

**FIGURE 2:**
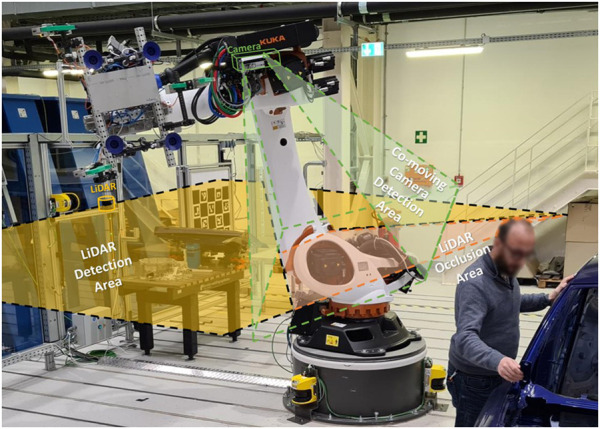
Proposed flexible and efficient sensor system.

**FIGURE 3:**
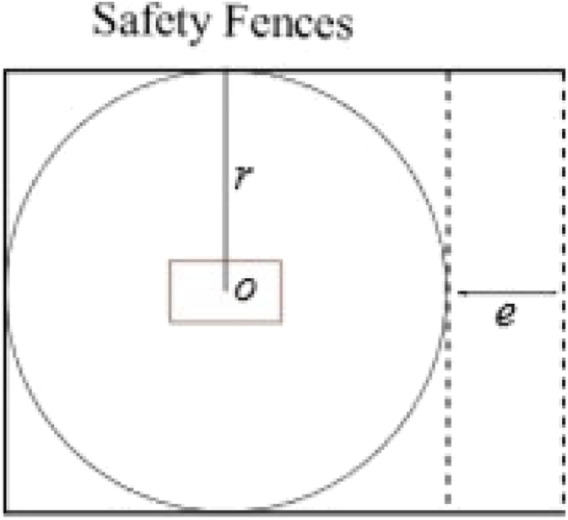
Simplified co-existence cell.

**FIGURE 4:**
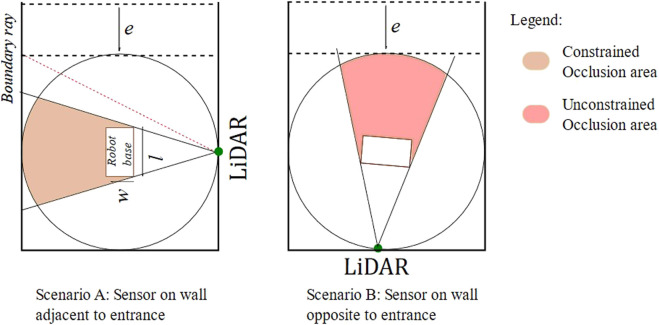
Different types of occlusions.

**FIGURE 5:**
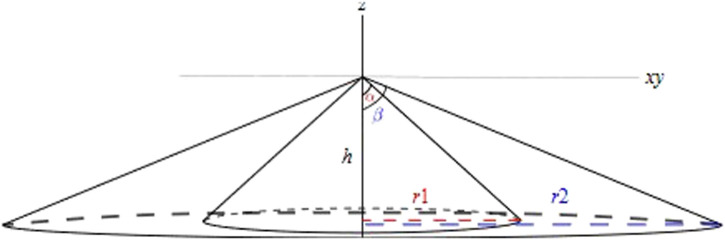
LiDAR sensor concept.

**FIGURE 6:**
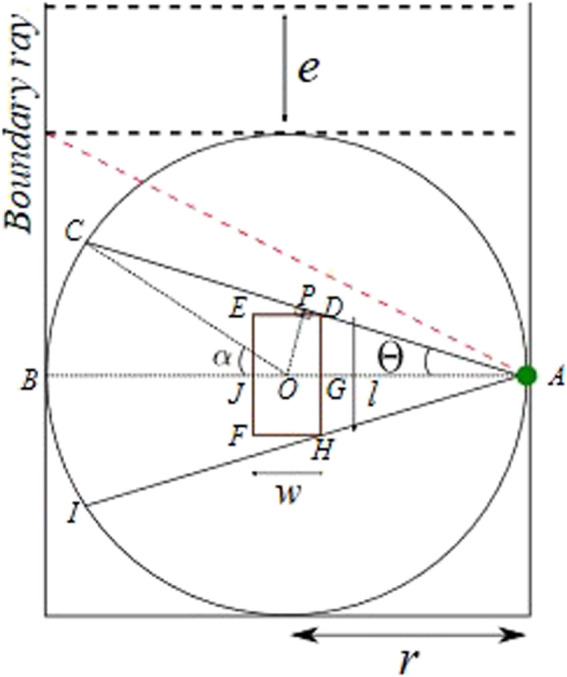
Constrained occlusion with sensor at middle of fence.

**FIGURE 7:**
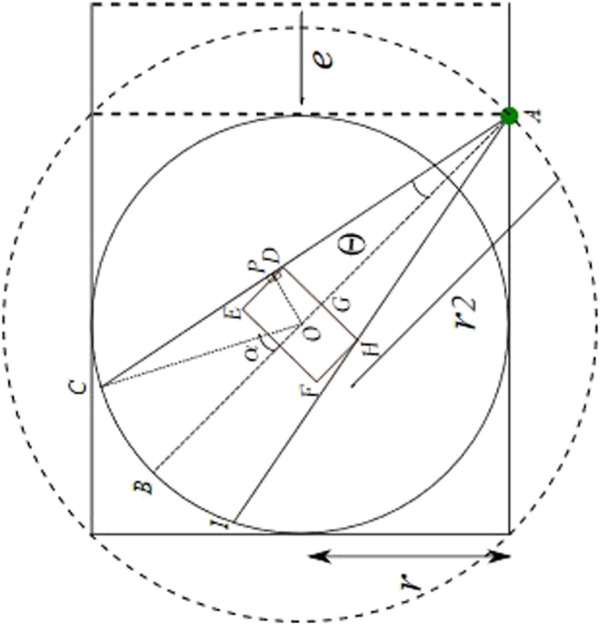
Constrained occlusion with sensor at the entrance.

**FIGURE 8:**
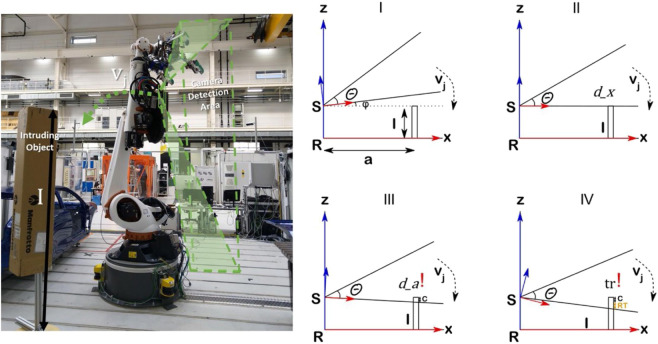
Data Capturing experiment for safety parameter estimation.

**FIGURE 9:**
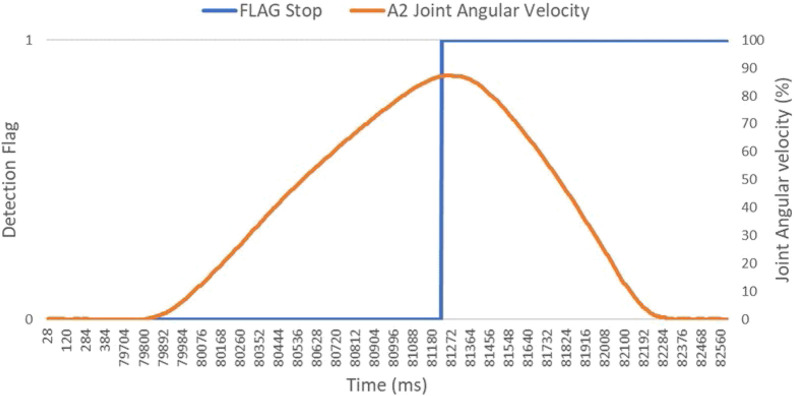
Complete trajectory for collision sensing and stop trigger for a single iteration.

**FIGURE 11:**
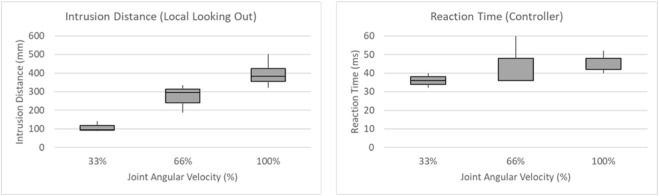
Box plot on multiple iterations at multiple robot velocities.

**FIGURE 12:**
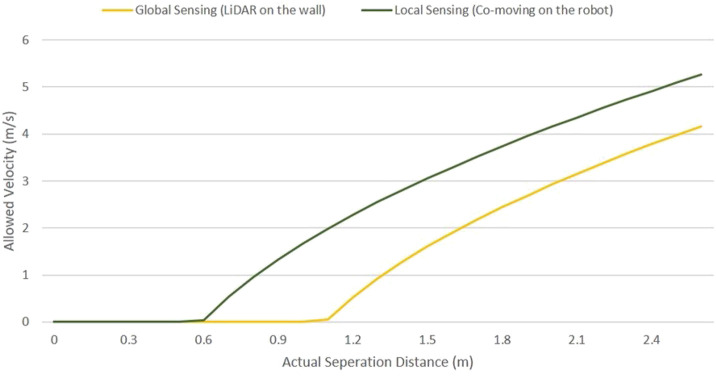
Safety distance and robot velocity for local (co-moving) and global collision sensing.

